# Premenstrual Syndrome Symptomatology among Married Women of Fertile Age based on Methods of Contraception (Hormonal versus Non-Hormonal Methods of Contraception)

**DOI:** 10.5539/gjhs.v6n2p105

**Published:** 2013-12-09

**Authors:** Nour Mohammad Bakhshani, Mohsen Hosseinbor, Zahra Shahraki, Nahid Sakhavar

**Affiliations:** 1 Children and Adolescents^’^ Health Research Center - Zahedan University of Medical Sciences, Zahedan, Iran; 2 Zahedan University of Medical Sciences, Zahedan, Iran

**Keywords:** premenstrual syndrome, PMS, PMDD, contraception, hormones

## Abstract

**Introduction:**

Premenstrual syndrome (PMS) refers to the cyclic occurrence of a set of disturbing physical, emotional or behavioral alterations that are of sufficient severity to interfere with interpersonal relations and routine life. Normal variations in gonadal estrogen and progesterone lead to biochemical reactions in the brain, resulting in PMS symptoms. This study aims to investigate the prevalence of PMS and PMDD signs among married women of fertile age (MWFA) based on the methods of birth control.

**Method and Materials:**

In a descriptive study, a number of 400 married women referring to 20 family healthcare clinics that use contraceptive methods were recruited and PMS questionnaire were administered to them.

**Results:**

From 400 subjects, 205 took oral contraceptive pills (hormonal methods of contraception) and 195 used other birth control methods (non-hormonal method). A number of 345 subjects (86.25%) at least experienced one PMS symptom and 55 subjects (13.75%) did not report any symptoms. Of those who use hormonal contraceptives (HCs), 182 (88.8%) reported PMS symptoms and 23(11.2) lacked any symptoms.

**Conclusion:**

About 86% of the subjects showed moderate to severe of PMS symptoms. Although using hormonal contraceptive method can theoretically reduce PMS symptoms, such effect was not observed in this study. The results of this research should be generalized with caution. Future studies are suggested.

## 1. Introduction and Background

Premenstrual disorders are a set of physical and psychological symptoms that are observed about a week before menses and decrease as the menstruation starts (C. S. Brown, Ling, Andersen, Farmer, & Arheart, 1994). When such symptoms are not severe, the term premenstrual syndrome (PMS) is used; however, if the symptoms are intense, premenstrual dysphoric disorder (PMDD) applies (Freeman, 2003). According to American College of Obstetricians and Gynecologists (ACOG), PMS is diagnosed when at least one moderate to severe emotional or physical symptom is present (ACOG, 2000). Based on the diagnostic criteria in the DSM-IV (APA, 2000), diagnosis of PMDD requires at least five psychological and physical symptoms, of which at least one should be a severe psychological Symptom.

Prevalence of PMS and PMDD as well as the prevalence of their symptoms based on the diagnostic criteria in the population under study (e.g., ethnicity and culture) have been reported differently (Anson, 1999; Sternfeld, Swindle, Chawla, Long, & Kennedy, 2002). More than 200 symptoms have been cited for PMS (Henderson, 2000) which are associated with poor quality of life and decreased efficiency in workplace (Dean, Borenstein, Knight, & Yonkers, 2006). Although the etiology of such disorders is not known exactly, it is thought that sex steroids, which are produced by ovarian corpus luteum, can produce PMS symptoms. Progestogens and progesterone with estrogen can lead to PMS symptoms. Moreover, the brain response systems contributing to PMS are serotonin and GABA systems. Progesterone metabolites, particularly allopregnanolone, act through the GABA system. Allopregnanolone has an effect similar to those of benzodiazepines, barbiturates, and alcohols (Backstrom et al., 2003). Based on the hypotheses related to the etiology of PMS/PMDD, that is thought that SSAIs and the compounds that prevent ovulation, such as gonadotropin-releasing hormone agonists (gnrh-As), seem to be effective treatments for this disorder (Backstrom et al., 2003). Moreover, there have been varying reports on the effects of hormonal contraception methods, such as OCPs, on women suffering from PMS (Backstrom et al., 2003; Bäckström, Hansson-Malmström, Lindhe, Cavalli-Björkman, & Nordenström, 1992; C. Brown, Ling, & Wan, 2001; Calhoun, 2012; Dennerstein et al., 1985; Ford, Lethaby, Mol, & Roberts, 2006; Freeman et al., 2001; Graham & Sherwin, 1992; Jarvis, Lynch, & Morin, 2008; Lopez, Kaptein, & Helmerhorst, 2012; Magill, 1995; Vanselow, Dennerstein, Greenwood, & de Lignieres, 1996; K. Wyatt, Dimmock, Jones, Obhrai, & O’Brien, 2001; K. M. Wyatt, Dimmock, Ismail, Jones, & O’Brien, 2004).

Hormonal treatment in PMS involves manipulation of the hormones proposed to be responsible for Development of PMS symptoms (Usman, Indusekhar, & O’Brien, 2008). Dennerstien et al. (1985) confirmed beneficial effects of progesterone on the both mood and physical symptoms of PMS but some studies failed to show effectiveness of progesterone (Sampson, 1979; Van der Meer, Benedek-Jaszmann, & Van Loenen, 1983). Findings of a review (Ford et al., 2006) revealed that effectiveness of progesterone in treatment of PMS differs based on participants, rout of administration, duration and doses. Patients with PMS treated by using oral contraceptive (OC) containing combination of drospirenone and ethinyl estradiol show more improvement compared with those treated with placebo, and symptom reductions were significant in acne, appetite and food craving (Freeman et al., 2001). It is thought that contraceptive agents that prevent ovulation should be effective for reducing PMS symptoms but there isn’t sufficient evidence to conclude hormonal contraceptives used by women reduce experiencing PMS symptoms.

Taking into account the absence of related research on Iranian women and the varying patterns of PMS disorders prevalence in different cultures and ethnicities, on the one hand, and inconsistencies regarding the effects of hormonal contraceptives (HCs) on PMS and PMDD symptoms, on the other hand, this study was conducted to examine the prevalence of PMS symptoms among MWFA referring to Zahedan’s healthcare centers that offer hormonal contraception methods as well as on those who used other (non-hormonal) contraceptive methods.

## 2. Method and Materials

In an analytical, descriptive study, from among the women referring to Zahedan’s family healthcare clinics who used oral contraceptive pills (OCPs) or other birth control methods, a number of 400 subjects aged 20-45 were selected. Of those selected, 205 used HCs for at least six months and the remaining 195 used other contraception methods such as IUD, condoms, calendar-based or natural contraceptive methods. For the sampling purpose, first a number of 20 healthcare centers were randomly selected. Then, participants recruited from the referrers based on the number of women covered by each center. Reproductive age-Women that met the following inclusion criteria were recruited: a) have used a method of contraception during past six months. b) Had not a history of mental disorder. C) Have lived in zahedan city at least for one year. We, also, did not include women who have changed their contraceptive method from hormonal to non-hormonal contraceptive method or conversely during past six months. This research has been verified by the research council of Zahedan University of Medical Sciences.

PMS questionnaire (Bakhshani, Mousavi, & Khodabandeh, 2009), which records patients’ demographics as well as their physical and psychological PMS symptoms was used to assess PMS symptoms. Participants were asked to fill the questionnaire based on their last three menses.

## 3. Results

Data on 400 participants, including 205 using HCs and 195 using non- HCs show that 345 participants (86.2%) have experienced at least one of the 21 PMS symptoms (88.8% HC_S_ users and 83.6% non- HCs users). In both groups, backache (with severity of moderate or severe) has been the most frequently reported symptom (38.6% of HCs users; 54.3% of non- HCs users). The overall prevalence of this somatic symptom was 47.7% (Table 1).

**Table 1:** Prevalence of PMS symptoms among married women by methods of contraception (N=400)

Symptoms		Using hormonal Contraception (n=205)		Using non- hormonal Contraception (n=195)		Total (N=400)	
		Mild F(%)	Moderate to Severe F(%)	Mild F(%)	Moderate to Severe F(%)	Mild F(%)	Moderate to Severe F(%)
**Psychological symptoms**	Depressed Mood	53(25.9)	35(17.2)	29(14.9)	33(16.9)	82(20.5)	68(17)
	Worry and Anxiety	34(16.6)	35(17.9)	42(21.5)	69(17.2)	99(24.7)	57(27.8)
	Felt suddenly Sad/Tearful	33(16.1)	44(21.4)	24(12.3)	33(16.9)	57(14.2)	77(19.2)
	Anger/Interpersonal conflicts	26(12.7)	40(19.5)	22(11.3)	22(11.3)	48(12)	62(15.5)
	Avoided Social activities and relationships	18(8.8)	33(16.1)	17(8.7)	14(7.2)	35(8.7)	47(11.7)
	Overwhelmed or out of control	26(12.7)	30(14.7)	39(20)	30(15.3)	65(16.2)	60(15)
	Change in appetite	35(17.1)	57(27.8)	40(20.5)	54(27.7)	75(18.7)	111(27.7)
	Hypersomnia/Insomnia	43(21)	62(30.3)	35(18.2)	61(31.3)	78(19.7)	122(30.7)
	Difficulty concentration	22(10.7)	25(12.2)	24(12.3)	14(7.2)	46(11.5)	39(9.7)
	Tiredness or Lethargic	62(30.2)	79(38.5)	62(31.8)	78(40)	124(31)	157(39.2)
**Somatic symptoms**	Breast pain or Swelling	43(21)	33(16.1)	35(17.9)	29(14.9)	78(19.5)	62(15.5)
	Headache	54(26.3)	73(35.6)	42(21.5)	68(34.9)	96(24)	141(35.2)
	Joint or Muscle pain	45(22)	62(30.3)	27(13.8)	41(21.1)	72(18)	103(25.7)
	Urinate frequency	28(13.7)	38(18.7)	18(9.2)	36(18.4)	46(11.5)	74(18.5)
	Weight gain	19(9.3)	21(10.3)	12(6.2)	14(7.2)	31(7.7)	35(8.7)
	Backache	43(21)	85(41.5)	37(19)	106(54.3)	80(20)	191(47.7)
	Acne	15(7.3)	17(8.3)	22(11.3)	14(7.2)	37(9.2)	31(7.7)
	Extremity swelling	18(8.8)	11(5.4)	14(7.2)	6(3.1)	22(5.5)	17(4.2)
	Nausea or Vomiting	40(19.5)	24(11.8)	30(15.4)	19(9.8)	70(17.5)	43(10.7)
	Abdominal bloating	21(10.2)	18(8.8)	21(10.8)	24(12.3)	42(10.5)	42(10.5)
	Dysfunction in social or economic performance	13(6.3)	15(7.4)	7(3.6)	10(5.1)	20(5)	25(6.2)

In addition, findings of current study indicate that the most prevalent psychological symptoms among women who used HCs are tiredness and lethargy (38.5%) followed by sleep disorders (30.3%), change in appetite (27.8%), anxiety and stress (27.8%). In the second group, the order of prevailing symptoms is almost similar to the first group: tiredness and lethargy (40%), sleep disorders (31.3%), change in appetite (27.7%), anxiety and stress (21.5 %).

Findings on the physical (somatic) symptoms among those who used HCs show that the most prevalent somatic symptoms are backache (41.5%), headache (35.6%), and joint or muscle pain (30.3%). The most frequent physical symptoms in the non-HCs users are backache (54.3%), headache (34.9%), and joint or muscle pain (21.1%). Compared to non HCs, prevalence rates of several symptoms (anxiety, sadness, interpersonal conflicts, difficulty in concentration, join or muscle pain and weight gain) in HCs users are higher than those in non- HCs users(see Table 1). Table 2 shows frequency of PMS by methods of contraception, 88.8% of women using HCs and 83.6% of women using non-HCs suffer from PMS. The percentage of participants with PMS do not differ by methods of contraception (Chi-Square =2.27, P =0.131). Also 8.78% of HCs users and 4.1% non-HCs users fulfilled criteria of PMDD (Table 3). Although the percentage of PMDD is higher in HCs users than non-HCs users but There is not a significant relationship between method of contraception and PMDD (Chi-Square =3.59, P =0.0578).

**Table 2:** Frequency of PMS based on methods of contraception

Methods of Contraception	F(%)		Total
	With PMS	Without PMS	
Using HCs	182(88.8)	23(11.2)	205
Using Non-HCs	163(83.6)	32(16.4)	195
Total	345	55	400

Chi-Square= 2.27, P = 0.131

**Table 3:** Frequency of PMDD based on methods of contraception

Methods of Contraception	F(%)		Total
	With PMDD	Without PMDD	
Using HCs	18 (8.78)	187(91.2)	205
Using Non-HCs	8(4.1)	187(95.9)	195
Total	26	374	400

Chi-Square= 3.59, P = 0.0578

## 4. Discussion

Aiming at determining the prevalence of PMS among 400 MWFA who used hormonal and non-hormonal contraceptives, this study showed that 345 subjects (86.2%) experienced at least one moderate to severe PMS symptom and 55 subjects (13%) did not show any symptoms.

Of MWFA who used HCs, 182 (88.8%) showed PMS symptoms and 23 (11.2%) did not. On average, 163 participants (83.6%) who used non- HCs reported PMS symptoms. About 6% participants in our study reported moderate to severe dysfunction in social or economic performance (7.4% HCs users and 5.1% non- HCs users). Current findings on the prevalence of PMS symptoms are somewhat lower but comparable to the results reported by other researches (Bakhshani et al., 2009; Chang, Holroyd, & Chau, 1995; Cleckner-Smith, Doughty, & Grossman, 1998; Thu & Diaz, 2006a).

Chung et al. (1995) reported 95% of Chinese women suffered from PMS symptoms. One study conducted by Cleckner-Smith et al. (1998) revealed that all of the subjects (n=78) showed at least one slight, 88% and 73% had moderate and sever symptom respectively.

Thu and Diaz (2006b) found that over 98% of respondents in Thailand suffered from one or more PMS symptom(s). Digueli et al. (1994) showed that PMS prevalence among Brazilian women was 8-86% depending on the investigated symptoms. In their study on 300 students aged 18-27, Bakhshani et al. (2009) found that 98.2% experienced at least one PMS symptom, the most prevalent of which were fatigue (84%), depression (72.3%), anxiety (70%), backache (69%), sleep disorders (66%). These prevalence rates are higher than findings of current study.

Digueli et al. (1998) found no relation between the use of hormonal medicine and PMS symptoms. In addition, one study on Brazilian women by Silva et al. revealed that women who have not used any hormonal contraceptives reported higher prevalence of the PMS that not consistent with our finding. Sadler et al. (2010) found that premenstrual symptoms were less common in those women currently using hormonal contraceptives compared with those not using any form of hormonal contraception. Also, a recent survey of US military women showed that low prevalence of premenstrual symptoms in users of injectable progestins. In summary, evidence from several studies (Coffee, Kuehl, Willis, & Sulak, 2006; Hourani, Yuan, & Bray, 2004; Sadler et al., 2010; Sulak, 2005; Coffee et al., 2006) suggest that hormonal contraceptive methods that suppress ovulation and may have a role in preventing or decreasing premenstrual symptoms.

Although it is thought that hormonal changes in corpus luteum are associated with PMS and hormonal manipulation can reduce PMS symptoms. Progesterone and estrogen together can lead to PMS symptoms and their severity highly dependent on estrogen dosage (Freeman, Rickels, Schweizer, & Ting, 1995). However, more investigations are required to find evidence supporting the idea that estrogen plays an obvious role in reducing PMS whereas progesterone lacks such capability and it even results in increased PMS symptoms.

In summary, despite the popularity of using hormonal contraceptives to manage PMS symptoms, the current study suggests that not only prevalence rates of PMS symptoms in HCs users are not lower than non- HCs users but also some symptoms (such as anxiety, anger, avoided social activities, difficulty concentration, join or muscle pain and weight gain) are more prevalent among HCs users. Due to several limitations of the study results should be generalized with caution:

A - Participants were recruited from patients referred to health centers rather than the community.
B - Previous experiences of women related to contraceptive methods may influence their current behaviors and tendency.
C - Those that prefer hormonal methods than non- hormonal methods may have different psychological characteristics.

Given the discussed issues and the findings of other researches, it can be concluded that despite the expectation that the use of HCs would abate PMS symptoms because of the estrogen content, but based on results of present study there is no beneficial effect on reducing PMS symptoms for using HCs. Therefore, controlled and prospective studies are suggested to examine the effects of hormonal manipulation in decreasing or increasing PMS symptoms. Moreover, considering the prevalence of PMS in both study groups in current study and considering findings of previous researches on risk factors of PMS (Johnson, 1987; Marvan, Diaz-Erosa, & Montesinos, 1998; Potter, Bouyer, Trussell, & Moreau, 2009; Tschudin, Bertea, & Zemp, 2010) that demonstrated impact of psycho-socio-cultural factors on PMS, it is suggested that sociocultural and psychobiological factors that can be involved in prevalence of PMS symptoms in societies should be identified. It is also recommended that proper training be given to women for controlling and dealing with PMS symptoms and for improving their lives during their childbearing period. In addition the required treatment and counseling facilities should be furnished to them.
